# Enhancing the Permeate Flux of Direct Contact Membrane Distillation Modules with Inserting 3D Printing Turbulence Promoters

**DOI:** 10.3390/membranes11040266

**Published:** 2021-04-07

**Authors:** Hsuan Chang, Chii-Dong Ho, Yih-Hang Chen, Luke Chen, Tze-Hao Hsu, Jun-Wei Lim, Chung-Pao Chiou, Po-Hung Lin

**Affiliations:** 1 Department of Chemical and Materials Engineering, Tamkang University, Tamsui, New Taipei 251, Taiwan; nhchang@mail.tku.edu.tw (H.C.); yihhang@mail.tku.edu.tw (Y.-H.C.); s8900511@gmail.com (T.-H.H.); a0937420623@gmail.com (C.-P.C.); ben225588@hotmail.com (P.-H.L.); 2 Department of Water Resources and Environmental Engineering, Tamkang University, Tamsui, New Taipei 251, Taiwan; luke@mail.tku.edu.tw; 3 Department of Fundamental and Applied Sciences, HICoE-Centre for Biofuel and Biochemical Research, Institute of Self-Sustainable Building, Universiti Teknologi PETRONAS, Seri Iskandar 32610, Perak Darul Ridzuan, Malaysia; junwei.lim@utp.edu.my

**Keywords:** permeate flux, DCMD module, 3D printing turbulence promoter, eddy promoter

## Abstract

Two geometric shape turbulence promoters (circular and square of same areas) of different array patterns using three-dimensional (3D) printing technology were designed for direct contact membrane distillation (DCMD) modules in the present study. The DCMD device was performed at middle temperature operation (about 45 °C to 60 °C) of hot inlet saline water associated with a constant temperature of inlet cold stream. Attempts to reduce the disadvantageous temperature polarization effect were made inserting the 3D turbulence promoters to promote both the mass and heat transfer characteristics in improving pure water productivity. The additive manufacturing 3D turbulence promoters acting as eddy promoters could not only strengthen the membrane stability by preventing vibration but also enhance the permeate flux with lessening temperature polarization effect. Therefore, the 3D turbulence promoters were individually inserted into the flow channel of the DCMD device to create vortices in the flow stream and increase turbulent intensity. The modeling equations for predicting the permeate flux in DCMD modules by inserting the manufacturing 3D turbulence promoter were investigated theoretically and experimentally. The effects of the operating conditions under various geometric shapes and array patterns of turbulence promoters on the permeate flux with hot inlet saline temperatures and flow rates as parameters were studied. The distributions of the fluid velocities were examined using computational fluid dynamics (CFD). Experimental study has demonstrated a great potential to significantly accomplish permeate flux enhancement in such new design of the DCMD system. The permeate flux enhancement for the DCMD module by inserting 3D turbulence promoters in the flow channel could provide a maximum relative increment of up to 61.7% as compared to that in the empty channel device. The temperature polarization coefficient ($\tau_{temp}$) was found in this study for various geometric shapes and flow patterns. A larger $\tau_{temp}$ value (the less thermal resistance) was achieved in the countercurrent-flow operation than that in the concurrent-flow operation. An optimal design of the module with inserting turbulence promoters was also delineated when considering both permeate flux enhancement and energy utilization effectiveness.

## 1. Introduction

Membrane distillation (MD) has technical feasibility advantages in separation and water treatment with the high permeate flux and low energy consumption. In an MD desalination process [1,2], the existence of temperature differences was built up by the hydrophobic membrane surface temperatures that always contact both hot and cold bulk temperatures. The need for a better temperature driving-force gradient is attracting global attention due to the vapor pressure difference for the permeate passing through the hydrophobic membrane with considerable heat loss. The significant thermal resistance occurs on the thermal boundary layer close to the membrane surface of saline feed stream, which creates a temperature gradient between both membrane surfaces and results in heat loss [3]. Moreover, a higher temperature polarization coefficient yielding augmentation of driving-force temperature gradients of both bulk feed streams comes out with the increment of trans-membrane permeate flux [4,5]. Development of desalination techniques with a higher permeate flux has been conducted for improved seawater desalination system [6,7]. Despite major advances in developing membrane materials on distillation performance [8,9,10] and pressure driven membrane separation processes are still facing the problems of the temperature polarization effect [11,12]. Previous studies in improving the permeate flux enhancement by incorporating proper flow alteration configurations such as filament [13,14,15], roughened surface [16] and eddy promoter [17] into the flow channel of DCMD modules to boost the turbulence intensity were also investigated. Having spacer filaments in the feed channel has been proven to dynamically change the thermal boundary layer and mitigate temperature polarization. It is well recognized that the turbulence promoters were added individually to the flow channel to generate vortices in enhancing the local shear stress on the membrane surface and to create secondary flows or eddies in the feed stream. Those previous studies confirmed that the mathematical model is capable of predicting heat and mass transfer behaviors in the MD desalination system compared to the experimental results. This study showcases a new design of 3D printing turbulence promoters by machining for generating turbulent intensity in the flow channel of DCMD modules. Extending previous studies, the present work focuses on the overall heat-transfer resistance in which the potential investigation of combining different geometric shapes of 3D turbulence promoters and various array patterns enhance hydrodynamic conditions. The 3D printing turbulence promoters present a higher designing flexibility so as to create multi-faceted geometric shapes under various scales with respect to traditional fabricating techniques [18,19], and this 3D printing technology was used to design spacer-filled flow channel in the membrane separation module to augment the device performance [20,21]. However, the permeate flux enhancement is associated with power consumption increments due to the augmented turbulent intensity attributed to inserting 3D turbulence promoters into the flow channel. The ratio of permeate flux enhancement to energy consumption increment was also assessed to indicate an optimal operation of considering economic feasibility.

Two kinds of 3D printing turbulence promoters, say circle and square shapes, were designed and fabricated to produce secondary flow characteristics and thus alleviate the temperature polarization effect in membrane distillation devices. The turbulence promoters inserted into the flow channel provided a larger convective heat-transfer coefficient due to disrupting the thermal boundary layer on the hot feed stream when compared with that of the device with empty channel. The objective of this study is to fabricate two geometric shapes turbulence promoters and stick onto the membrane surface of the flow channel in hot saline water stream to reduce its temperature polarization effect owing to reducing the heat transfer resistance, and thus, a higher permeate flux was achieved. A theoretical model validated by the experimental data was also developed to predict the pure water productivity for comparisons under various operating conditions, and the fluid velocity distribution was also presented using Ansys software (2018).

## 2. Experimental Setup and Materials

**Figure 1:**
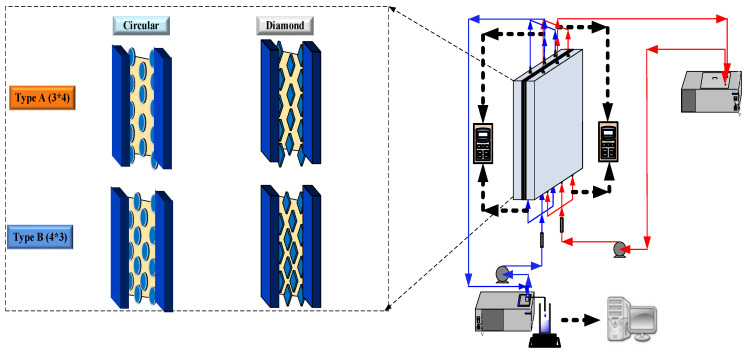
Schematic diagram of the experimental setup for the direct contact membrane distillation (DCMD) system.

**Figure 2:**
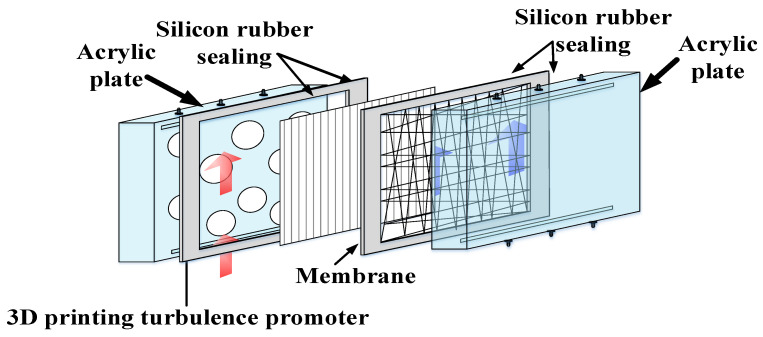
Fabrication structure of the DCMD module.

**Figure 3:**
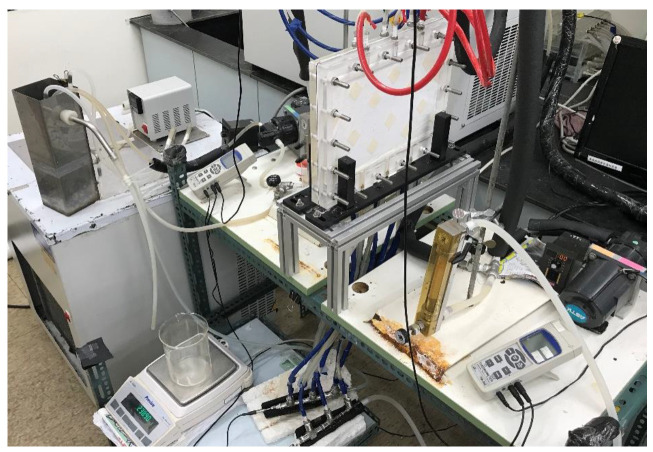
A photo of the experimental setup.

**Figure 4:**
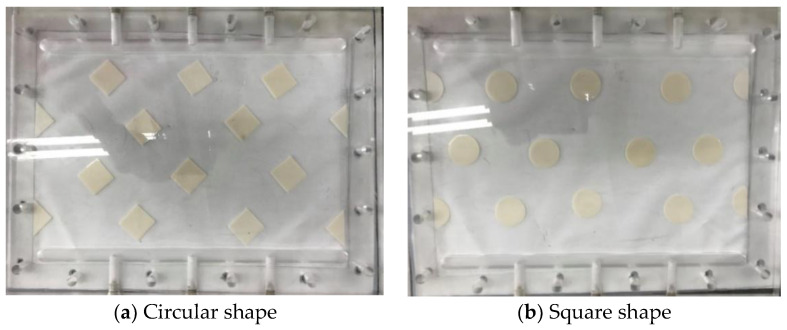
Top view of two kinds of turbulence promoters.

The schematic detailed configuration and the fabrication structure of the flat-plate DCMD module are illustrated in Figure 1 and Figure 2, respectively, and a photo of the present experimental setup is shown in Figure 3 with acrylic plates as outside walls on a parallel-plate channel. The DCMD module contains two flow channels with inserting 3D turbulence promoters into the hot feed flow channel and empty channel of the cold stream. The empty channel is constructed with a 0.1 mm nylon fiber routed as a supporting material. The 3D printing turbulence promoters were constructed with a 1mm-thick and glued with Cyanoacrylate Adhesive (Chang Chun Plastics Co., Ltd., Taiwan) on the acrylic plate of the hot feed side in contact with the membrane surface acting as eddy promoters. The printing material of turbulence promoters was made with polyester elastomer and attached onto the hydrophobic membrane surface to offer the eddy motion around those obstacles. The length, width, and clearance of the permeate channel between the two plates were 21 cm, 29 cm, and 1 mm, respectively. The top view of two geometric shapes of turbulence promoters is specified in Figure 4a,b, in which the array pattern of the turbulence promoters was used as a configuration parameter.

Two parallel-plate flow channels (*L* = 0.21 m, *W* = 0.29 m, *d* = 1 mm) separated by a hydrophobic composite membrane made of PTFE/PP (polytetrafluoroethylene and polypropylene) with a nominal pore size of 0.1, a porosity of 0.72, and a thickness of 130 µm (ADVANTEC) were conducted the experimental run as the permeating medium in this study.

Between the hydrophobic composite membrane and the acrylic plate is inserting turbulence promoters into a 1 mm-thick silicon rubber which sealed and glued upon the acrylic plate to form a channel and to prevent leakage. Nylon fibers of 0.2 mm diameter was implemented to wind upon the hydrophobic membrane surface as a supporter grid in preventing from membrane bending and wrinkling. The artificial saline water of 3.5 wt% NaCl was prepared by adding inorganic salts NaCl into distilled water. The experiments were conducted for adjusting various inlet hot saline temperatures (40, 45, 50, 55 °C) regulated by the thermostat, and controlling various flow rates (0.1, 0.2, 0.3, 0.4 L/min) of both sides with using a rotameter while the temperature kept 25 °C of the cold stream. Comparisons were made of permeate fluxes under various operation conditions to study the device performances between two modules with/without inserting 3D printing turbulence promoters. The experimental run of permeate flux condensed in the cold stream side was then collected and weighted using an electronic balance to measure and record the amount of permeate flux on the PC.

## 3. Theoretical Formulations

### 3.1. Mass and Heat Transfer

**Figure 5:**
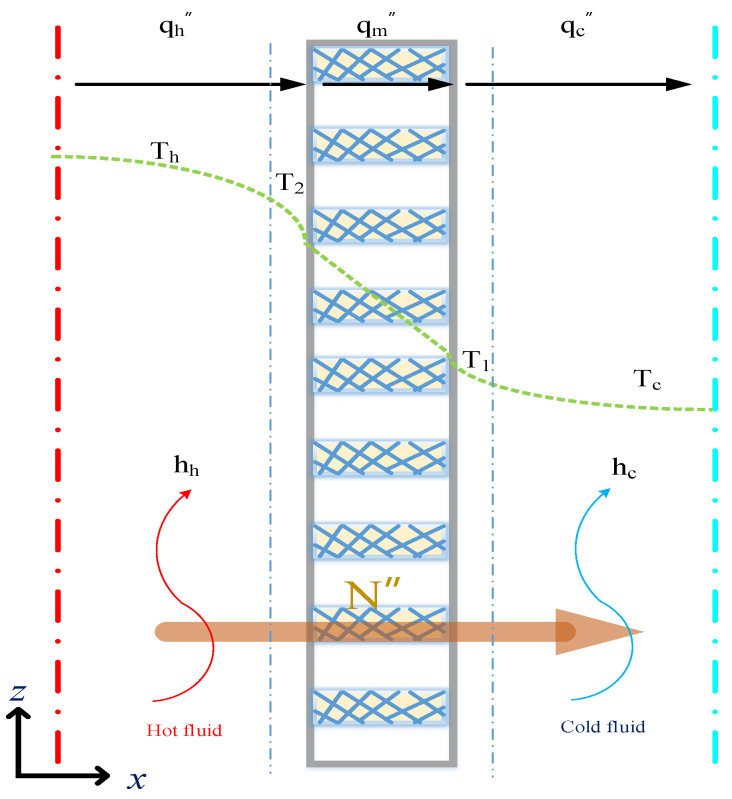
Mass transfer mechanisms and thermal boundary layer in the DCMD module.

A theoretical formulation coupling both heat and mass transfer mechanisms for the DCMD system, as shown Figure 5, to study how hot saline water was vaporized at the pore entrances of the membrane surface according to the vapor-water equilibrium, then vapor only diffused through porous hydrophobic membranes and condensed in the cold stream as pure water productivity. A mass transfer model is needed to describe the concentration of the hot saline water feed while the heat transfer model is required to determine the temperature gradients at each membrane surface due to the permeate flux transferring. Balances of permeate flux by mass diffusion and enthalpy flow conservation by heat conduction were formulated simultaneously as follows.

The membrane permeation coefficient ($c_{m}$) and the trans-membrane saturation vapor pressure difference (ΔP) have been used extensively in mass transfer analysis of permeate flux for membrane distillation processes [22,23]

(1) $$N^{\prime \prime }=c_{m}\Delta P=c_{m}\left[P_{2}^{sat}(T_{2})-P_{1}^{sat}(T_{1})\right]=c_{m}{\frac{dP}{dT}|}_{T_{m}}(T_{2}-T_{1})=c_{m}\frac{P_{m}\lambda M_{w}}{RT_{m}^{2}}(T_{2}-T_{1})$$

where the membrane permeation coefficient is the addition of Knudsen diffusion and Poiseuille flow.

(2) $$c_{m}={\left(\frac{1}{c_{K}}+\frac{1}{c_{M}}\right)}^{-1}={\left\{{\left[1.064\frac{\varepsilon r}{\tau \delta_{m}}{\left(\frac{M_{w}}{RT_{m}}\right)}^{1/2}\right]}^{-1}+{\left[\frac{1}{{\left|Y_{m}\right|}_{\ln}}\frac{D_{m}\varepsilon }{\delta_{m}\tau }\frac{M_{w}}{RT_{m}}\right]}^{-1}\right\}}^{-1}$$

The saturation vapor pressure ($P_{1}^{sat}$) of the hot fluid side can be estimated using water activity coefficient ($a_{w}$), which can be determined using a correlation,

(3) $$P_{1}^{sat}=y_{w}P=x_{w}a_{w}P_{w}^{sat}$$

(4) $$a_{w}=1-0.5x_{\mathrm{NaCl}}-10x_{\mathrm{NaCl}}^{2}$$

and the tortuosity (τ) can be estimated using the porosity of the membrane [8]

(5) τ=1/ε

Next, the heat balance of the DCMD module could be described on the principle of the non-isothermal process with making the energy balance of enthalpy flow conservation, and the simultaneous occurrences of heat and mass transfer in the DCMD module in each heat transfer region (a) the hot saline water feed; (b) the hydrophobic composite membrane and (c) the cooling water. The energy balance equations were derived for each heat transfer region under the steady-state operation according to the schematic diagram of the DCMD module in Figure 5 and were shown in Equations (6)–(8) as follows:

(6) $${q^{\prime \prime }}_{h}=h_{h}(T_{h}-T_{2}\;)\;\mathrm{the}\;\mathrm{hot}\;\mathrm{saline}\;\mathrm{water}\;\mathrm{feed}\;\mathrm{region}\;$$

(7) $${q^{\prime \prime }}_{m}=N^{\prime \prime }\lambda +\frac{k_{m}}{\delta_{m}}(T_{2}-T_{1})\;\;\mathrm{the}\;\mathrm{membrane}\;\mathrm{region}$$

(8) $${q^{\prime \prime }}_{c}=h_{c}(T_{1}-T_{c}\;)\;\mathrm{the}\;\mathrm{cooling}\;\mathrm{water}\;\mathrm{region}\;$$

where $N^{\prime \prime }\lambda$ is characterized as the latent heat of vaporization and $k_{m}/\delta_{m}=h_{h}(T_{2}-T_{1}\;)$ is the conductive heat transfer, and the thermal conductivity of the membrane $k_{m}$ can be determined by the thermal conductivities of vapor in the membrane pore $k_{g}$ and the solid membrane material $k_{s}$ by Warner [24] as:

(9) $$k_{m}=\varepsilon \;k_{g}+(1-\varepsilon )k_{s}$$

The combinations of the heat flux term from Equations (1), (2) and (7) leads to the overall heat transfer coefficient of the membrane as follows:

(10) $$\begin{array}{c} {q^{\prime \prime }}_{m}=N^{\prime \prime }\lambda +\frac{k_{m}}{\delta_{m}}(T_{2}-T_{1})\;\\ =\left(c_{m}\frac{((1-x_{\mathrm{NaCl}})(1-0.5x_{\mathrm{NaCl}}-10x_{\mathrm{NaCl}}^{2})P_{2}+P_{1})\lambda^{2}M_{w}}{2RT_{m}^{2}}+\frac{k_{m}}{\delta_{m}}\right)(T_{2}-T_{1})=H_{m}(T_{2}-T_{1}) \end{array}$$

### 3.2. Temperature Polarization

The temperature polarization coefficient $\tau_{temp}$ is an indicator to reveal the magnitude of the thermal boundary-layer resistance which governs the permeate flux of pure water productivity, and is commonly used to express as the ratio of membrane surface temperatures’ gradient to bulk temperatures’ gradient as follows:

(11) $$\tau_{temp}=(T_{2}-T_{1})/(T_{h}-T_{c})$$

The one-dimensional mathematical treatments were developed among all heat transfer regions under steady-state operations of $q^{\prime \prime }={q^{\prime \prime }}_{h}={q^{\prime \prime }}_{m}={q^{\prime \prime }}_{c}$ with assuming well insulation on the outside of the DCMD module, as illustrated by the schematic diagram in Figure 5 Both membrane surface temperatures ($T_{1}$ and $T_{2}$) and the convective heat-transfer coefficients ($h_{h}$) were obtained by equating Equations (6) and (10) (${q^{\prime \prime }}_{h}={q^{\prime \prime }}_{m}$) and Equations (8) and (10) (${q^{\prime \prime }}_{m}={q^{\prime \prime }}_{c}$), respectively, as follows:

(12) $$T_{h}=T_{2}+\frac{H_{m}}{h_{h}}\left(T_{2}-T_{1}\right)$$

(13) $$T_{c}=T_{1}-\frac{H_{m}}{h_{c}}\left(T_{2}-T_{1}\right)$$

Then, a more simplified form of $\tau_{temp}$ expressed in terms of the heat transfer coefficient

(14) $$\tau_{temp}=\frac{h_{h}h_{c}}{h_{h}h_{c}+h_{h}H_{m}+h_{c}H_{m}}$$

**Figure 6:**
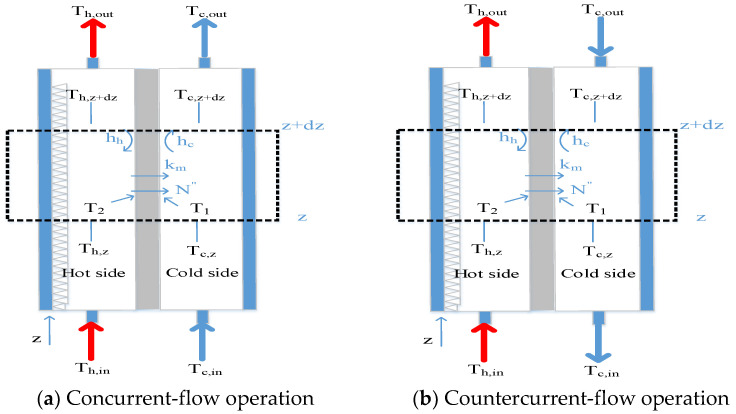
The energy balance made within a finite fluid element (concurrent-flow operations).

The procedure for calculation of theoretical values of the heat transfer coefficient will be described as follows. First, with the given operation conditions, the heat transfer coefficient is determined from Equations (12) and (13). Next, with the known inlet and outlet temperatures of both hot and cold streams, a temporary value of $T_{1}$ (or $T_{2}$) is estimated from Equation (12) once $T_{2}$ (or $T_{1}$) is assumed in Equation (13). Further, the convective heat-transfer coefficient is calculated from Equation (1). With this calculated value of the convective heat-transfer coefficient, new values of $T_{1}$ and $T_{2}$ are then re-calculated from Equations (12) and (13). If the calculated values of $T_{1}$ and $T_{2}$ are different from the assumed values, continuous calculation by iteration is needed until the last assumed values of membrane surface temperatures meet the finally calculated values. The modeling equations of the energy balances were obtained by making the energy-flow diagram presented in a finite fluid element, as shown in Figure 6, to solve the temperature distributions of both hot and cold stream sides as

(15) $$\frac{dT_{h}}{dz}=\frac{-q^{\prime \prime }W}{Q_{h}\rho_{h}C_{p,h}}=\frac{-W}{Q_{h}\rho_{h}C_{p,h}}H_{m}\tau_{temp.}(T_{h}-T_{c})$$

(16) $$\frac{dT_{c}}{dz}=\frac{q^{\prime \prime }W}{Q_{c}\rho_{c}C_{p,c}}=\frac{W}{Q_{c}\rho_{c}C_{p,c}}H_{m}\tau_{temp.}(T_{h}-T_{c})\;\mathrm{Concurrent}-\mathrm{flow}\;\mathrm{operation}$$

(17) $$\frac{dT_{c}}{dz}=\frac{-q^{\prime \prime }W}{Q_{c}\rho_{c}C_{p,c}}=\frac{-W}{Q_{c}\rho_{c}C_{p,c}}H_{m}\tau_{temp.}(T_{h}-T_{c})\;\mathrm{Countercurrent}-\mathrm{flow}\;\mathrm{operation}\;$$

The calculated convective heat-transfer coefficients were conveyed to solve the temperature distributions of both hot and cold stream sides in two simultaneous ordinary differential equations of Equations (15) and (16) for concurrent-flow operation (or Equation (17) for countercurrent-flow operation) by marching the fourth order Runge-Kutta method along the length of the module.

### 3.3. Enhancement Factor

The 3D printing turbulence promoters are inserted in the conduit of hot feed stream side instead of using the device of empty channel. The enhancement factor $\alpha^{p}$ depending on the geometric shapes and array patterns was corrected to calculate the augmented convective heat transfer coefficients in DCMD modules with inserting the 3D printing turbulence promoters and were carried out an iterative procedure as [25,26,27]

(18) $$\alpha^{p}=Nu^{p}/Nu_{lam}$$

where

(19) $$Nu^{p}=\frac{h_{h}D_{h,h}}{k}\;3D\;\mathrm{printing}\;\mathrm{turbulence}\;\mathrm{promoters}\;\mathrm{module}$$

(20) Nulam=4.36+0.036RePr(Dh,h/L)1+0.011(RePr(Dh,h/L))0.8 empty channel module 

The 3D printing turbulence promoters inserted into flow channel play a significant role as the eddy promoter, and a better interpretation of both heat and mass transfer behaviors in the turbulent flow could be described by a new method based on dimensional analysis of the Buckingham’s π theorem. The Nusselt number of flow channels with inserting 3D printing turbulence promoters can be related to dimensionless groups:

(21) Nup=f(Wpd,Re,Pr)

where $W_{p}$ and $D_{h,h}$ are the average equivalent width of 3D printing turbulence promoters and hydraulic diameter of the hot stream side, respectively.

### 3.4. Power Consumption Increment

The power consumption increment is unavoidable due to inserting 3D printing turbulence promoters into the hot saline water flowing channel as eddy promoters. The power consumption only the friction losses to walls of both hot and cold streams were significant and may be determined using Fanning friction factor $f_{F}$,

(22) $$\begin{array}{c} \;H_{i}={\dot{m}}_{h}\ell w_{f,h}+{\dot{m}}_{c}\ell w_{f,c}=Q_{h}\rho_{h}\ell w_{f,h}+Q_{c}\rho_{c}\ell w_{f,c}\\ \;i=promoter,empty\; \end{array}$$

(23) $$\ell w_{f,j}=\frac{2f_{F,j}{\bar{v}}_{j}^{2}L}{d_{h,i}},\;j=h,c$$

The average velocity and equivalent hydraulic diameter of cold and hot stream sides are defined as follows:

(24) $${\bar{\nu }}_{h}=\frac{Q_{h}}{\left(dW-D_{p}W_{p}n_{p}\right)},{\bar{\nu }}_{c}=\frac{Q_{c}}{dW}\;$$

(25) $$D_{h,c}=\frac{4\left(dW\right)}{2\left(d+W\right)};\;D_{h,h}=\frac{4A}{P}=\frac{4(dW-D_{p}W_{p}n_{p})}{\left[2(d+n_{p}D_{p})+2(W-n_{p}W_{p})\right]}$$

(26) Reh=ρhv¯h2dh,hμh; Rec=ρcv¯c2dh,cμc

The Fanning friction factor can be estimated using a correlated equation based on channel’s aspect ratio (β=d/W),

(27) fF,j=24(1−1.3553β+1.9467β 2−1.7012β 3+0.9564β 4−0.2537β 5)/Rej,j=h,c

The relative extents $I_{P}$ of power consumption increment was illustrated by calculating the percentage increment in the device with inserting turbulence promoters, based on the device of empty channel as

(28) $$I_{P}=\frac{H_{promoter}-H_{empty}}{H_{empty}}\times 100\%$$

where the subscripts promoter and empty represent the channels with inserting turbulence promoters and the empty channel.

### 3.5. The Design of 3D Printed Turbulence Promoters

**Figure 7:**
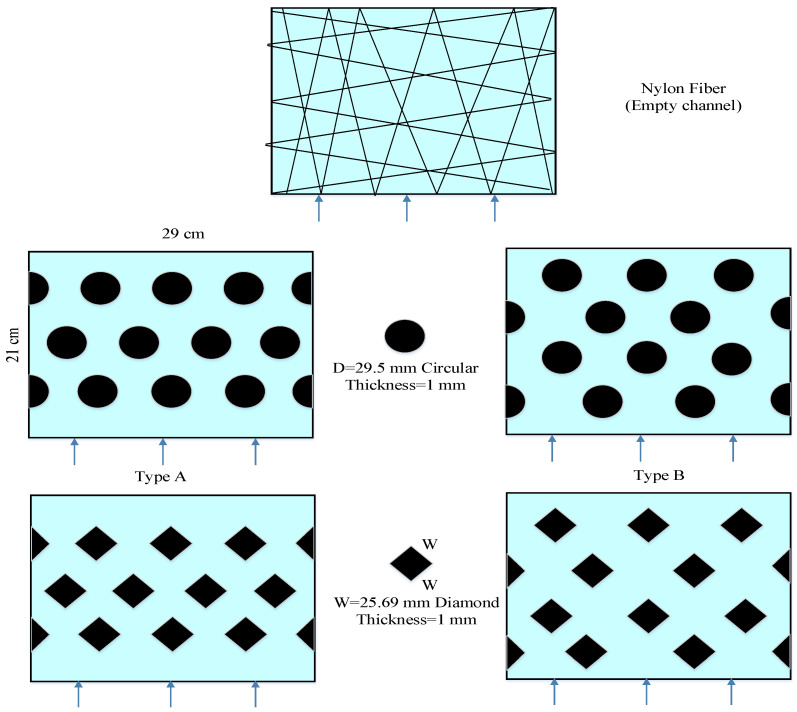
A schematic diagram of three flow channel types.

A smart attempt to advance hydrodynamic conditions in pressure-driven membrane separation processes with gluing 3D printing turbulence promoters on the membrane surface can accomplish the goal of very economical process in terms of implementing cost and speed. The 3D printing technology presents a higher flexibility in designing various complex geometric shapes compared to traditional manufacturing techniques [28]. This is one of the main advantages of 3D printing processes to produce different shapes of turbulence promoters [19] in precisely tailoring the 3D geometric shapes through a layer-by-layer machining process. Two types of turbulence promoters were designed as circular and square by 3D printing technology and empty channel (wound with nylon fiber), respectively, and inserted in the flowing channel throughout the experiments, as shown in Figure 7 Circular type was made of a circle with a diameter of 29.5 mm. Square type was made of a square with each length of 25.69 mm, all of the turbulence promoters had the same area and volume. The height of flowing channel and turbulence promoters are both 1 mm. The turbulence promoters were printed by a 3D printer (ATOM 2.5EX, Taiwan) in making three dimensional solid objects from a digital file of different shapes and incorporated into the flow path and glued to the membrane surface. The printing filament was fabricated from polyester elastomer to durable and hydrophobic promoter structures. Two kinds of array patterns with four half shape and ten full shape turbulence promoters were installed in the hot stream side. Since the printed promoter icon occupied 13% coverage of the entire membrane permeate area, therefore the effective permeate area of the membrane in blocking vapor flux is taken into account in calculation procedure.

### 3.6. CFD Simulation

**Figure 8:**
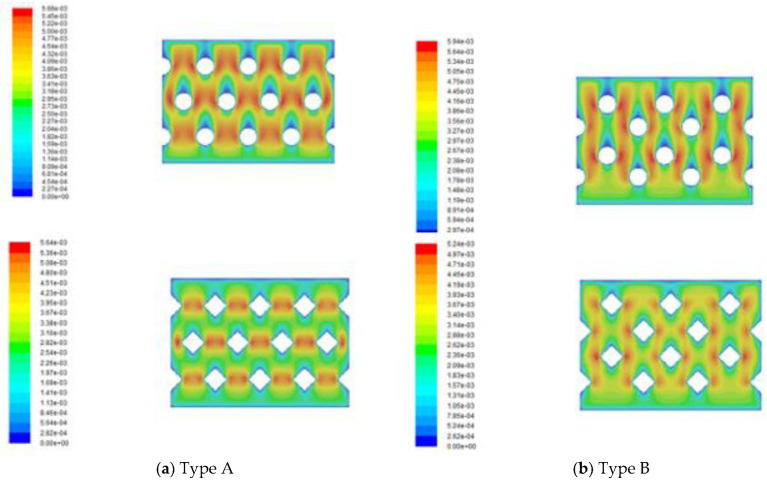
Simulation result of velocity profiles in different arrangements of turbulence promoters.

The detail of the 3D printing protocol of the fabricated promoters is included in Figure 7 The permeate flux area was 6.09 × 10^−2^ mA commercial CFD solver was used to solve the velocity profile of the Navier-Stokes equations for the incompressible flow coupled using the CFD solver algorithm under steady state and the volume of fluid model. The convergence criteria were the residuals of continuity and velocity below 10^−^The geometry of the flow channel and 3D mesh were illustrated using Ansys software (2018). Hexahedral elements were appropriate for this structure including a wedge element. The mesh sizes were approximately 78334, 77900, 73361 and 72433 for each type of turbulence promoters with skewness of 0.8, respectively. The density and viscosity for the fluid was assumed to be 1019 kg/m^3^ and 1.06 cp, respectively. The inlet velocity was given by calculating the volumetric flow rate and the inlet cross area, and the distribution of corrected velocity was obtained from CFD simulation with conserving the voidage change due to inserting turbulence promoters, as shown in Figure 8.

### 3.7. Numerical Scheme

The temperatures on both sides of membrane surfaces ($T_{1}$ and $T_{2}$) were calculated by equating Equations (6) and (10) and Equations (8) and (10) with the initial guess of the convective heat-transfer coefficient ($h_{h}$) until the iteration procedure reaches the convergence with 0.1% error of accuracy control and validated by the experimental flux ${N^{\prime \prime }}_{\exp}$ under the known inlet and outlet temperatures of both stream sides. The estimated values of membrane surfaces and the convective heat-transfer coefficient were used to solve Equations (13) and (14) (Equation (15) for countercurrent-flow operations) numerically using the fourth order Runge-Kutta method along the flowing direction of DCMD module, and thus, the theoretical permeate flux and permeate flux enhancement were obtained. The temperature distributions were predicted theoretically not only in the hot/cold bulk flows but also on the membrane surfaces of both hot and cold feed sides under concurrent- and countercurrent-flow operations, respectively. Comparisons were made for the permeate flux of the channel with inserting turbulence promoters and the empty channel under both concurrent- and countercurrent-flow operations.

## 4. Results and Discussion

### 4.1. Flux Enhancement by Inserting Turbulence Promoter in DCMD Module

**Figure 9:**
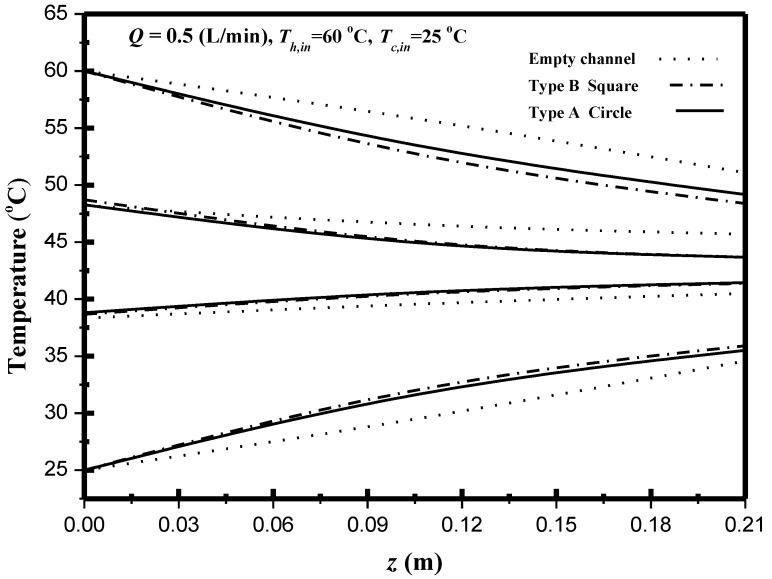
Effect of geometric turbulence promoters on temperature profiles.

The permeate flux is dependent on the temperature gradient between the membrane surface $T_{1}$ and $T_{2}$ in the DCMD system. The effect of the geometric turbulence promoters on the longitudinal temperature profiles of both channels in the DCMD module was shown in Figure 9. The devices with inserting turbulence promoters with various geometric shapes and array patterns for eddy promoting come out with temperature polarization reduction. Both membrane-surface and bulk temperatures trend taper along the flowing direction in concurrent-flow operations with decreasing the driving-force temperature gradients shown in Figure 9 while the driving-force temperature gradients in countercurrent-flow operations keep a relative higher average value than those in concurrent-flow operations, and thus, the permeate flux enhancement in countercurrent-flow operations was accomplished.

Reduction of the temperature polarization effect was achieved using eddy promoters in flow channel for a favorable result investigated by computational simulation [29]. The heat transfer coefficients of inserting turbulence promoters in flow channel expressed by Nusselt number and determined by the Buckingham’s π theorem, were correlated with the experimental data. The enhancement factor $\alpha^{p}$ derived from the correlation of Nusselt number for the channel with inserting turbulence promoters was determined via a regression analysis as:

(29) $$\alpha^{P}=\frac{Nu^{P}}{Nu_{lam}}=0.72\ln\;{\left(\frac{W_{P}}{D_{h}}\right)}^{2.015}$$

**Figure 10:**
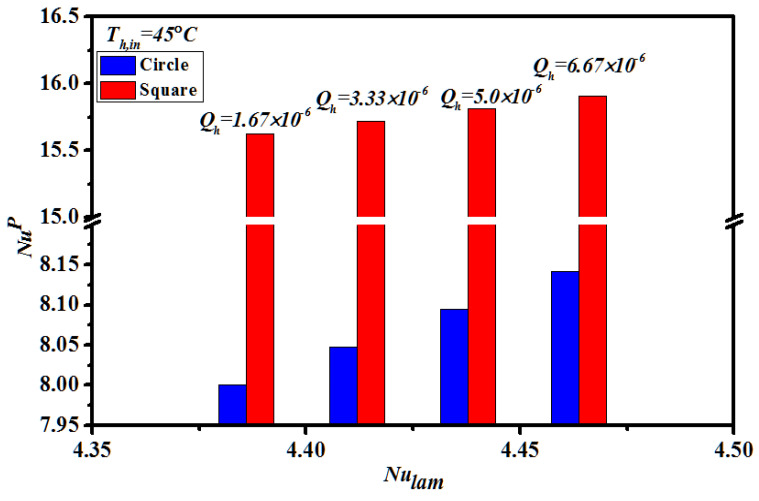
Comparisons of theoretical Nusselt numbers for the channels with inserting two geometric shapes and Type A of turbulence promoters.

The Nusselt numbers for the empty channel are in linear diagonal straight line with the experimental data, as shown in Figure 10, while the correlation expression of Nusselt numbers is also applicable for the channel with inserting two geometric shapes and array patterns of turbulence promoters. The Nusselt number in the channels with inserting turbulence promoters is higher than that of the empty channel.

Inserting turbulence promoters interrupts the thermal boundary layer on the membrane surface that diminishes heat transfer resistance; hence the permeate flux was boosted. The permeate fluxes were calculated through the enhancement factor, Equation (27), for predicting the Nusselt number, as referred to the heat transfer efficiency, with inserting turbulence promoters in flow channel. A relative increase of permeate flux $I_{E}$ was used to make comparisons between the permeate flux of the channel with turbulence promoters and the empty channel.

The performance of permeate flux enhancement $I_{E}$ was illustrated by calculating the percentage increase in the device with inserting turbulence promoters, based on the device of empty channel as

(30) $$I_{E}=\frac{{N^{\prime \prime }}_{promoter}-{N^{\prime \prime }}_{empty}}{{N^{\prime \prime }}_{empty}}\times 100\%$$

The comparisons have shown that the increased $I_{E}$ for the channel with inserting turbulence promoters of two geometric shapes and two array patterns in both concurrent- and countercurrent-flow operations. In general, the permeate flux enhanced by the inserting turbulence promoters is more significant in countercurrent-flow operations than those in concurrent-flow operations. As expected, either the increase of hot feed flow rate or the use of countercurrent-flow operations of turbulence promoters results in more permeate flux. The theoretical permeate flux for various hot feed flow rates and inlet temperatures under concurrent- and countercurrent-flow operations are summarized in Table 1 and Table 2, respectively. It is also seen from Table 1; Table 2 that the order of the performance of permeate flux enhancement for the device inserting turbulence promoters is the following: Type B Square > Type B Circle > Type A Circle > Type A Square. The Square turbulence promoter with Type B array patterns enhances the permeate flux enhancement by approximately 20–40% compared to Type A, whereas the Circle turbulence promoters of Type A shows a lower permeate flux enhancement than that in Type B by approximately 5–10%, under the same operation conditions. Overall, inserting turbulence promoters into flow channel shows a great potential to significantly enhance the permeate flux in the DCMD module. Thus, temperature polarization reduction is crucial not only in pressure-driven membrane distillation processes but also in other water treatment technologies.

Velocities and vortices in enhancing the local shear stress on the membrane surface to create secondary flows or eddies in the feed stream are two factors to affect the permeate flux enhancement of the device inserting square and circle turbulence promoters. The average equivalent width of square and circle turbulence promoters is $9.72\times 10^{-3}\;m$ and $1.22\times 10^{-2}\;m$, respectively, and hence the velocities in the device with inserting square turbulence promoters are larger than those in the circle ones. Besides, the device with inserting geometric shapes of square turbulence promoters in flowing channel generates more intensive vortices and eddies than those in the device with inserting circle turbulence promoters, resulting the square turbulence promoter is better than circle.

**Figure 11:**
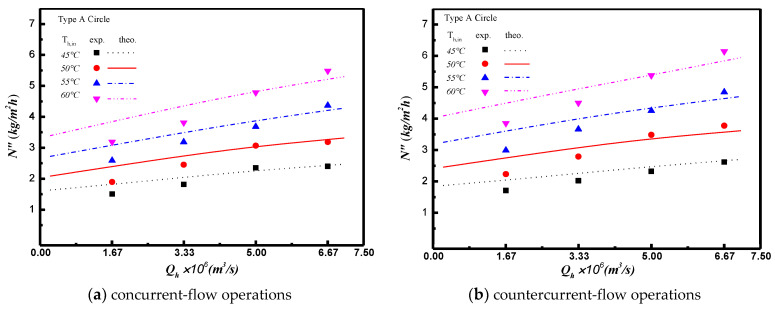
Effects of saline water flow rate and flow pattern on permeate flux for Type A circle turbulence promoter.

**Figure 12:**
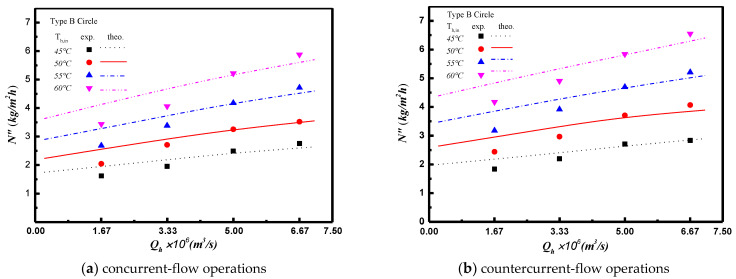
Effects of saline water flow rate and flow pattern on permeate flux for Type B Circle turbulence promoter.

The permeate flux enhancement with inserting turbulence promoters in flow channel is illustrated in Figure 11 and Figure 12 for both experimental results and the theoretical predictions. Two shapes and two array patterns of the inserting turbulence promoters produces the higher turbulence intensity that results in the higher heat transfer or, the higher permeate flux. Comparisons of theoretical predictions of permeate flux were made for both concurrent- and countercurrent-flow operations as depicted in Figure 11 and The agreement of the simulation results with those obtained from experimental runs is pretty good, as indicated in Figure 11 and As expected, either the increase of hot saline water flow rate or the countercurrent-flow operation results in more permeate flux. A relative increase of permeate flux $I_{E}$ was used to compare the permeate flux of the channel with inserting turbulence promoter to the empty channel. In general, the permeate flux enhanced by inserting turbulence promoters is more significant in countercurrent-flow operations than that in concurrent-flow operations.

**Figure 13:**
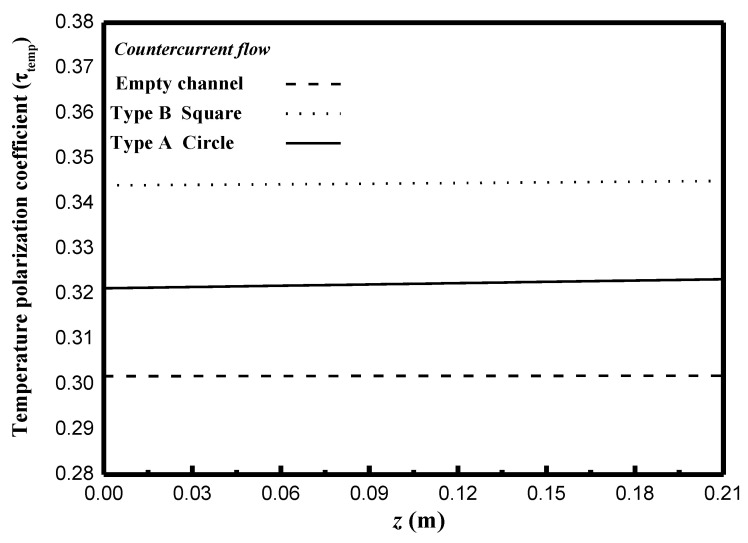
Effects of geometric shapes on $\tau_{temp}$ of countercurrent-flow operations.

This study also measured, predicted and compared the effects of geometric shapes of turbulence promoters on temperature polarization for countercurrent-flow operations, as depicted in Figure 13. Inserting turbulence promoters into the hot feed stream results in a higher $\tau_{temp}$ value (a lesser temperature polarization) was found due to the reduction of the thermal boundary-layer thickness as compared to the $\tau_{temp}$ value of the module without inserting turbulence promoters, as shown in Figure 13. In addition, a larger $\tau_{temp}$ value (the less thermal resistance) was achieved in the countercurrent-flow operation than that in the concurrent-flow operation.

### 4.2. Analysis of Cake Properties

The precision index of experimental uncertainty of each individual measurement of permeate flux is calculated as referred to Moffat [30] directly from the experimental runs as follows:

(31) $$S_{N^{\prime \prime }}={\left\{\sum_{i=1}^{N_{\exp}}\frac{{\left({N^{\prime \prime }}_{\exp}-{N^{\prime \prime }}_{theo}\right)}^{2}}{N_{\exp}-1}\right\}}^{1/2}$$

and the uncertainty of the reproducibility of the permeate fluxes will be associated with the mean precision index.

(32) $$S_{\bar{N^{\prime \prime }}}=\frac{S_{N^{\prime \prime }}}{\sqrt{N_{\exp}}}$$

The mean precision index of the experimental measurements of permeate flux enhancement was evaluated within $8.12\times 10^{-3}\leq S_{\bar{N^{\prime \prime }}}\leq 2.20\times 10^{-2}$ for both concurrent-flow and countercurrent-flow operations. The permeate flux’s experimental results prove the theoretical predictions’ validity by defining the accuracy between the numerical solutions and the experimental results as follows:

(33) $$E(\%)=\frac{1}{N}\sum_{i=1}^{N_{\exp}}\frac{\left|{N^{\prime \prime }}_{theo}-{N^{\prime \prime }}_{\exp}\right|}{{N^{\prime \prime }}_{\exp}}\times 100$$

where ${N^{\prime \prime }}_{theo}$ indicates the theoretical prediction of permeate flux while $N_{\exp}$ and ${N^{\prime \prime }}_{\exp}$ are the number of experimental measurements and the experimental data of permeate flux. The error analysis of the experimental measurements determined by Equation (33) for both the theoretical prediction is 0.11≤E≤9.68.

**Figure 14:**
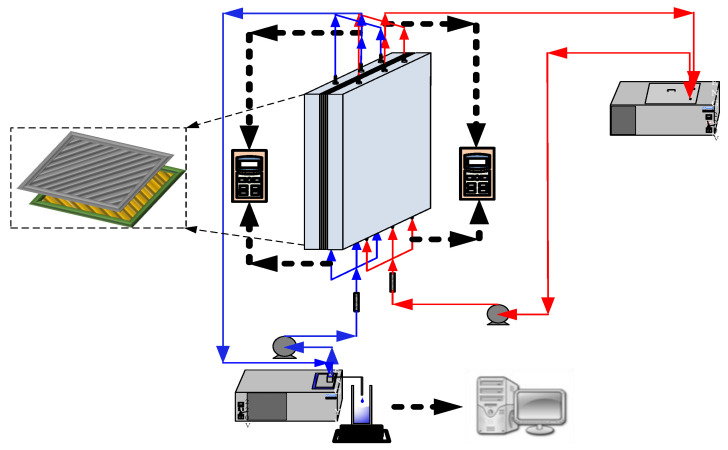
Schematic diagram of the experimental setup for the DCMD system of the channels with inserting diagonal carbon-fiber spacers.

**Figure 15:**
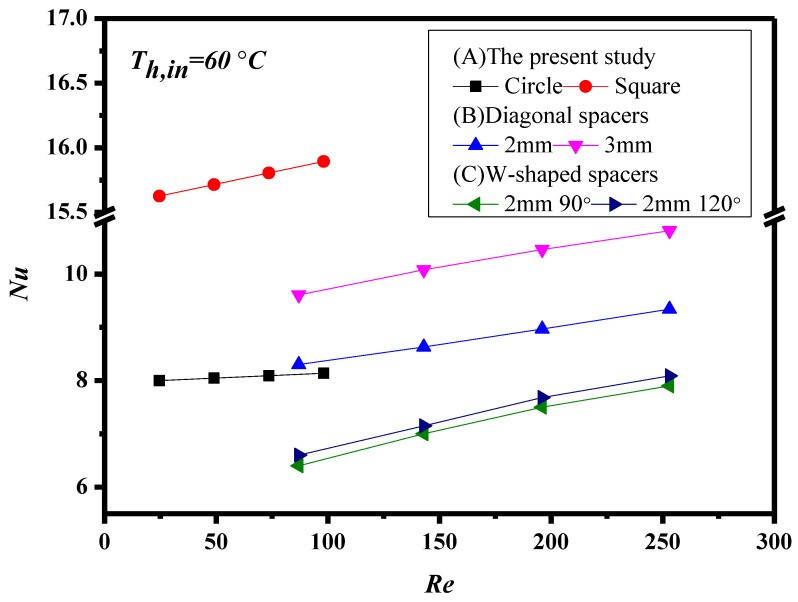
Comparisons of theoretical Nusselt numbers of the channels with inserting 3D turbulence promoters and two type of carbon-fiber spacers (countercurrent flow).

The present work extends the existing study except for inserting 3D turbulence promoters instead of W-shaped carbon-fiber spacers [31]. Besides, to perform additional DCMD tests with the canals filled with supporting mesh, the experiment runs were conducted the channels of DCMD devices with inserting diagonal carbon-fiber spacers to replace the W-shaped carbon-fiber spacers, as shown in Figure 14. Therefore, the graphical representation for comparisons with theoretical predictions of Nusselt numbers obtained in the present study, diagonal carbon-fiber spacers and W-shaped carbon-fiber spacers [31] illustrates why the present design of inserting 3D turbulence promoters is preferred, presented by Figure 15 for countercurrent-flow operations. This is the value and originality of the present study regarding to the implementing cost and technical feasibility.

### 4.3. Energy Consumption

**Figure 16:**
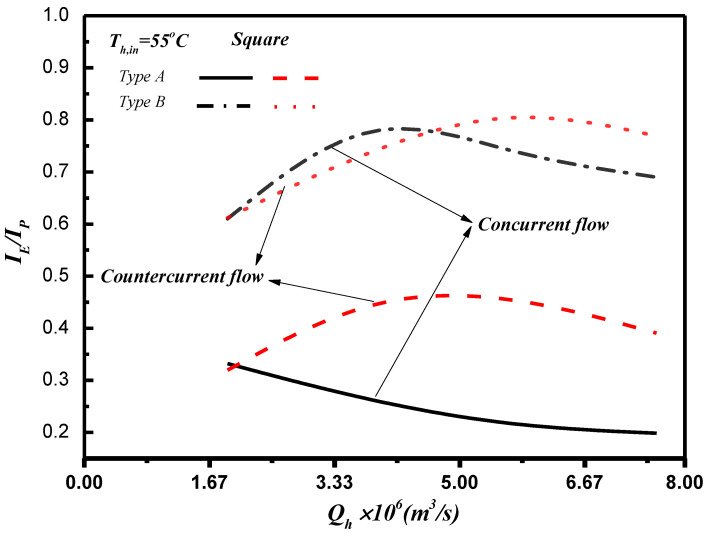
Effects of flow patterns on $I_{E}/I_{P}$.

This study further evaluated the device design’s effectiveness by considering the ratio of permeate flux increment to power consumption increment $I_{E}/I_{P}$ (Equations (28) and (30)) due to the flow resistance with more power consumptions caused by the inserting turbulence promoters in the flow channel. The effect of feed flow rate, two array patterns of square turbulence promoters, inlet hot feed temperature, and concurrent-/countercurrent-flow operations on $I_{E}/I_{P}$ are illustrated in Figure 16.

The 3D printing turbulence promotor inserted in the flow channel is the aim to achieve the augmented turbulence intensity in lessening temperature polarization layers and amplify the convective heat-transfer coefficient as well, and thus, the permeate flux is boosted. The desirable permeate flux improvement and the undesirable power consumption increment are two conflict effects encountered in making economic consideration by inserting turbulence promoters in the flow channel. The percentage of permeate flux enhancement is less than that of energy consumption increment results in a lower value of $I_{E}/I_{P}$, which indicates that power consumption increment cannot compensate more permeate flux due to a limited vapor transporting rate through the membrane by the convective heat-transfer coefficient in the hot stream side for the device with inserting turbulence promoters. In other words, the array pattern of Type B gives higher value of $I_{E}/I_{P}$, which reflects at the expenses of energy consumption are more effective in increasing the permeate flux. Running countercurrent-flow operations with a larger temperature gradient between hot saline feed and cold side than the concurrent-flow operations gives the higher $I_{E}/I_{P}$ value. The comparison reveals that the countercurrent-flow operation obtains permeate flux more effectively than that in the concurrent-flow operation.

As the $I_{E}/I_{P}$ values decrease with the increasing hot feed flow rate larger than $5.0\times 10^{6}{\;m}^{3}/s$ for both concurrent- and countercurrent-flow operations and array patterns of square turbulence promoters, one may notice that there are existing an optimal $I_{E}/I_{P}$ values. Notice that the $I_{E}/I_{P}$ ratio of the countercurrent-flow operation is higher than that of the concurrent-flow operation. Therefore, comparisons on both operations of flow patterns and array patterns were made on considering both the effective utilization of power consumption relative to the increase of permeate flux to indicate the trend of economic feasibility with inserting turbulence promoters for some specific hot feed rates in this study.

The introduction of inserting 3D turbulence promoters has positive effects on the heat and mass transfer rate, and outcome assessments of permeate flux improvements requires determining some key operating parameters, such as flow rates, geometric shapes, array patterns and flow patterns. The decrease in temperature polarization (A larger $\tau_{temp}$) with inserting 3D turbulence promoters is accomplished in tapering thermal boundary layers, and hence, establishing a larger trans-membrane mass transfer across the hydrophobic membrane due to the driving-force temperature gradients increment. Meanwhile, a suitable selection of the operating parameters for the device with inserting 3D turbulence promoters would be come out an evident advantage for practical applications with the economic sense which allows the specification setting by the designer. Actually, the present study is an extension of our previous work of DCMD module [31] to apply the device with inserting 3D turbulence promoters instead of carbon-fiber spacers. Though the phenomenon of heat and mass transfer behaviors in the present study could be analogized from those in our previous work [31], the manners of velocity and thermal boundary layers are somewhat different in heat and mass transfer mechanisms, and the implementing the experimental setup are somewhat easier and lesser fabricating cost.

## 5. Conclusions

A parallel-plate direct contact membrane distillation module with inserting 3D printing turbulence promoters to enhance the permeate flux was investigated theoretically and experimentally. The theoretical predictions of the permeate flux enhancement by inserting turbulence promoters were calculated and validated by experimental data, and the correlated expression of Nusselt number was obtained. Thorough comparisons of the permeate flux enhancement for various hot feed flow rate, inlet saline temperature, and various geometric promoter shapes and array patterns under both concurrent- and countercurrent-flow operations were examined. The comparisons of the permeate flux enhancement were drawn to the following conclusions:

1. The higher inlet hot feed temperature with the array pattern of Type B result in a higher permeate flux for both concurrent- and countercurrent-flow operations. A maximum of 61.7% permeate flux enhancement was found in the device with inserting turbulence promoters compared to that in the empty channel device.
2. The permeate flux is principally driven by the temperature gradient between both membrane surfaces along the flowing direction. A higher permeate flux is achieved in countercurrent-flow operations than that in concurrent-flow operations due to the larger temperature gradient for countercurrent-flow operations.
3. The ratio $I_{E}/I_{P}$ of permeate flux enhancement to power consumption increment was used to examine the economic viewpoint to increase pure water productivity in the present implementation. The economic consideration concluded that the power utilization is more effective for the channel with inserting turbulence promoters, and the optimal value of $I_{E}/I_{P}$ was obtained at some hot feed flow rate between $3.3\times 10^{6}{\;m}^{3}/s$ and $5.0\times 10^{6}{\;m}^{3}/s$.

In this paper, only the permeate flux enhancement and energy consumption increment were evaluated by inserting turbulence promoters into the saline feed channel. The alternative geometric shapes and array patterns of 3D printing turbulence promoters require further investigation.

## Figures and Tables

**Table 1:** Effects of geometric shapes and array patterns on flux enhancement for concurrent flow.

$T_{h,\;in}$ (°C)	$Q_{h}\times 10^{6}$ (m^3^ s^−1^)	Empty Channel	Circle				Square			
			Type A		Type B		Type A		Type B	
		${N^{\prime \prime }}_{theo}$ (kg m^−2^ h^−1^)	${N^{\prime \prime }}_{theo}$ (kg m^−2^ h^−1^)	$I_{E}$ (%)	${N^{\prime \prime }}_{theo}$ (kg m^−2^ h^−1^)	$I_{E}$ (%)	${N^{\prime \prime }}_{theo}$ (kg m^−2^ h^−1^)	$I_{E}$ (%)	${N^{\prime \prime }}_{theo}$ (kg m^−2^ h^−1^)	$I_{E}$ (%)
55	1.67	1.92	2.59	34.8	2.68	39.5	2.23	16.1	2.79	45.3
	3.33	2.35	3.19	35.7	3.38	43.8	2.65	12.7	3.80	61.7
	5.0	2.79	3.68	31.8	4.18	35.8	3.09	10.7	4.30	54.3
	6.67	3.25	4.37	34.4	4.72	45.2	3.58	10.1	4.91	51.2
60	1.67	2.41	3.19	32.0	3.44	42.7	2.72	12.8	3.49	44.8
	3.33	2.97	3.81	28.2	4.06	36.7	3.33	12.1	4.38	47.4
	5.0	3.50	4.79	36.8	5.22	49.1	4.21	20.2	5.50	57.2
	6.67	4.02	5.48	36.3	5.88	46.3	4.79	19.2	5.59	49.5

**Table 2:** Effects of geometric shapes and array patterns on flux enhancement for countercurrent flow.

$T_{h,\;in}$ (°C)	$Q_{h}\times 10^{6}$ (m^3^ s^−1^)	Empty Channel	Circle				Square			
			Type A		Type B		Type A		Type B	
		${N^{\prime \prime }}_{theo}$ (kg m^−2^ h^−1^)	${N^{\prime \prime }}_{theo}$ (kg m^−2^ h^−1^)	$I_{E}$ (%)	${N^{\prime \prime }}_{theo}$ (kg m^−2^ h^−1^)	$I_{E}$ (%)	${N^{\prime \prime }}_{theo}$ (kg m^−2^ h^−1^)	$I_{E}$ (%)	${N^{\prime \prime }}_{theo}$ (kg m^−2^ h^−1^)	$I_{E}$ (%)
55	1.67	2.26	2.99	32.3	3.18	40.0	2.61	15.4	3.28	45.1
	3.33	2.64	3.66	38.6	3.92	48.8	3.22	21.9	4.10	55.0
	5.0	3.06	4.25	38.8	4.70	52.5	3.73	21.9	4.94	61.0
	6.67	3.56	4.85	36.2	5.21	46.3	4.22	18.5	5.58	56.7
60	1.67	2.86	3.85	34.6	4.17	45.8	3.33	16.4	4.28	49.6
	3.33	3.28	4.50	37.1	4.90	49.3	3.91	19.2	5.07	54.5
	5.0	3.87	5.37	38.7	5.84	50.9	4.61	19.1	5.85	51.1
	6.67	4.41	6.14	39.2	6.55	48.5	5.36	21.5	6.87	55.7

## Abbreviations

$a_{w}$	Water activity in NaCl solution
Cp,c	Heat capacity of cold fluid (J kg^−1^K^−1^)
Cp,h	Heat capacity of hot fluid (J kg^−1^K^−1^)
$c_{m}$	Mass transfer coefficient of membrane (kg m^−2^ Pa^−1^ s^−1^)
$D_{h}$	Equivalent hydraulic diameter of empty channel (m)
$D_{h,h}$	Equivalent hydraulic diameter of hot side (m)
$D_{h,c}$	Equivalent hydraulic diameter of cold side (m)
d	Height of flow channel (m)
Dp	Height of 3D printed turbulence promoter (m)
E	Deviation of experimental results from the theoretical predictions
$f_{F}$	Fanning friction factor
$h_{c}$	Convection coefficient of cold fluid (W m^−2^ K^−1^)
$h_{h}$	Convection coefficient of hot fluid (W m^−2^ K^−1^)
$H_{i}$	Hydraulic dissipate energy (J kg^−1^), i=promoter,empty
$H_{m}$	Thermal convection coefficient of membrane (W m^−2^ K^−1^)
$I_{E}$	Raised percentage of permeate flux
$I_{P}$	Raised percentage of hydraulic loss
L	Axial distance (m)
k	Thermal conductivity coefficient of hot saline feed (W m^−1^ K^−1^)
kg	Thermal conductivity coefficient of the vapor in the membrane pore (W m^−1^ K^−1^)
ks	Thermal conductivity coefficient of the solid membrane material (W m^−1^ K^−1^)
ℓwf	Friction loss of conduits (J kg^−1^)
$M_{W}$	Molecular weight of water (kg mol^−1^)
$\dot{m}$	Mass flow rate (kg s^−1^)
$N^{\prime \prime }$	Permeate flux (kg m^−2^ h^−1^)
$Nu_{}$	Nusselt number
$Nu^{p}$	Nusselt number of the turbulence promoter
$Nu_{lam}$	Dimensionless Nusselt number for laminar flow
*n_p_*	Number of promoters per row
P	Pressure (Pa)
$P_{1}^{sat}$	Saturation vapor pressure in the cold feed flow side (Pa)
$P_{2}^{sat}$	Saturation vapor pressure in the hot feed flow side (Pa)
$P_{w}{}^{sat}$	Saturated vapor pressure of pure water (Pa)
Pr	Prandtl number
${q^{\prime \prime }}_{}$	Heat transfer rate (W/m^2^)
${q^{\prime \prime }}_{c}$	Heat transfer rate between cooling plate and cold fluid (W/m^2^)
${q^{\prime \prime }}_{h}$	Heat transfer rate between hot fluid and membrane surface (W/m^2^)
${q^{\prime \prime }}_{m}$	Heat transfer rate between membrane surface of hot fluid and air gap (W/m^2^)
Q	Volumetric flow rate (m^3^ s^−1^)
R	Gas constant (8.314 J mol^−1^ K^−1^)
*Re*	Reynolds number
$Sh_{lam}$	Dimensionless Schmidt number for laminar flow
$SN^{\prime \prime }$	The precision index of an experimental measurements of permeate flux (kg m^−2^ h^−1^)
$S\bar{N^{\prime \prime }}$	The mean value of $SN^{\prime \prime }$(kg m^−2^ h^−1^)
T	Temperature (°C)
$T_{m}$	Mean temperature in membrane (°C)
$\bar{v}$	Average velocity (m s^−1^)
Wp	Average equivalent width of 3D printed turbulence promoters
${\left|Y_{m}\right|}_{\ell n}$	Natural log mean mole fraction of air
$x_{NaCl}$	Liquid mole fraction of NaCl
$x_{w}$	Liquid mole fraction of water
z	Axial coordinate along flow direction (m)
*Greek letters*	
$\alpha^{p}$	Heat-transfer enhancement factor
β	Aspect ratio of the channel
ΔP	Vapor pressure difference of membrane (Pa)
$\delta_{m}$	Thickness of membrane (µm)
ε	Membrane porosity
λ	Latent heat of water (J/kg)
μ	Fluid viscosity (kg s^−1^ m^−1^)
ρ	Density (kg m^−3^)
$\tau_{temp}$	Temperature polarization coefficients
*Subscripts*	
1	Membrane surface on cold feed side
2	Membrane surface on hot feed side
*h*	In the hot feed flow channel
*c*	In the cold feed flow channel
*promoter*	Inserting turbulence promoters
*empty*	Inserting nylon fiber as supporters
*exp*	Experimental results
*in*	Inlet
*lam*	Empty channel
*out*	Outlet
*theo*	Theoretical predictions
*Superscripts*	
*p*	The channel with inserting turbulence promoters
